# APOE4 Alters Tau Propagation in Regions Associated with Early Alzheimer’s Disease in a Humanized Mouse Model of Tau Spread

**DOI:** 10.1002/alz.089837

**Published:** 2025-01-03

**Authors:** Hirra A Arain, Juan F Idiarte, Tsering D Lama, Helen Y Figueroa, Jia Guo, Karen E Duff, Tal Nuriel

**Affiliations:** ^1^ Department of Cell Biology and Pathology, New York, NY USA; ^2^ Columbia University Irving Medical Center, New York, NY USA; ^3^ Taub Institute for Research on Alzheimer’s disease, New York, NY USA; ^4^ Deparment of Cell Biology and Pathology, New York, NY USA; ^5^ Zuckerman Institute, Columbia University, New York, NY USA; ^6^ UK DRI at University College London, London United Kingdom

## Abstract

**Background:**

Possession of the APOE4 allele is the strongest genetic risk factor for developing the sporadic form of Alzheimer’s disease (AD). Studies investigating APOE4’s associated AD risk have largely centered on APOE4’s propensity to regulate the deposition of extracellular amyloid beta plaques. More recent attempts to characterize APOE4’s role in AD have brought into question the role APOE4 may possess in modulating the pathogenesis of intracellular tau tangles. However, to date, it remains unclear if APOE4 can alter pathological tau spread and if these effects are dependent on regions associated with early tau accumulation in AD.

**Method:**

To recapitulate tau spread observed early in AD, we developed a novel human APOE mouse that expresses misfolded human tau localizing to the entorhinal cortex (EC). These mice were generated by crossing homozygous mice possessing various humanized APOE alleles to mice harboring the human MAPT P301L mutation. Human MAPT was placed under control of the neuropsin‐tTa promoter to selectively expresses misfolded tau to the EC. Both male and female mice were analyzed at various ages up until 27 months using various in‐vivo and ex‐vivo techniques to measure tau pathology and propagation, spatial memory defects, and neurodegeneration.

**Result:**

We observed differential tau spread and neurodegeneration altered by APOE genotype in both a sex‐dependent and age‐dependent manner. Our results point predominantly to decreased tau pathology in regions associated with early AD in homozygous APOE4 mice. Analysis of the middle‐aged cohort revealed a significant decrease in tau propagation and associated atrophy to the hippocampus and lateral EC in homozygous female APOE4 mice. The observed effect on neurodegeneration and tau burden were attenuated in both male counterparts.

**Conclusion:**

Here we report the generation and characterization of a novel APOE‐tau mouse model that recapitulates the region‐specific tau pathogenesis observed in early AD. Our results offer novel insights into APOE4’s role in AD by altering the sex‐dependent propagation of pathological tau along anatomically connected, vulnerable AD brain regions.

